# DNA Methylation Modulates Nociceptive Sensitization after Incision

**DOI:** 10.1371/journal.pone.0142046

**Published:** 2015-11-04

**Authors:** Yuan Sun, Peyman Sahbaie, DeYong Liang, Wenwu Li, Xiaoyou Shi, Paige Kingery, J. David Clark

**Affiliations:** 1 Department of Anesthesiology, Stanford University School of Medicine, Stanford, California, United States of America; 2 Department of Anesthesiology, Veterans Affairs Palo Alto Health Care System, Palo Alto, California, United States of America; University of Kentucky Medical Center, UNITED STATES

## Abstract

DNA methylation is a key epigenetic mechanism controlling DNA accessibility and gene expression. Blockade of DNA methylation can significantly affect pain behaviors implicated in neuropathic and inflammatory pain. However, the role of DNA methylation with regard to postoperative pain has not yet been explored. In this study we sought to investigate the role of DNA methylation in modulating incisional pain and identify possible targets under DNA methylation and contributing to incisional pain. DNA methyltranferase (DNMT) inhibitor 5-Aza-2′-deoxycytidine significantly reduced incision-induced mechanical allodynia and thermal sensitivity. Aza-2′-deoxycytidine also reduced hindpaw swelling after incision, suggesting an anti-inflammatory effect. Global DNA methylation and DNMT3b expression were increased in skin after incision, but none of DNMT1, DNMT3a or DNMT3b was altered in spinal cord or DRG. The expression of proopiomelanocortin *Pomc* encoding β-endorphin and *Oprm1* encoding the mu-opioid receptor were upregulated peripherally after incision; moreover, *Oprm1* expression was further increased under DNMT inhibitor treatment. Finally, local peripheral injection of the opioid receptor antagonist naloxone significantly exacerbated incision-induced mechanical hypersensitivity. These results suggest that DNA methylation is functionally relevant to incisional nociceptive sensitization, and that mu-opioid receptor signaling might be one methylation regulated pathway controlling sensitization after incision.

## Introduction

Postoperative pain of moderate to severe intensity is experienced by 30–40% of patients after surgeries [1, 2]. Unrelieved postoperative pain is a common cause for unplanned hospital admission and contributes to suboptimal functional outcomes. Current therapeutic approaches to postoperative pain are limited by our narrow understanding of the underlying biological mechanisms. Therefore, examining the mechanisms involved in supporting pain after surgical incision would have significant value in addressing these problems.

Epigenetics refers to environmentally-supported changes in DNA and chromatin structure that do not alter DNA sequence. Such changes include the chemical modification of histone proteins, DNA methylation and microRNA expression [3]. DNA methylation is a covalent modification and involves the transfer of a methyl group to cysteine residues at CpG sites, which are 5'-CG-3' dinucleotide sequences in the genome, and clusters of CpG sites known as CpG islands, which are often in the promoter regions of genes [4]. This modification has been linked to many physiological and pathological processes, such as embryo development [5], aging [6], cancer [7, 8], psychiatric disorders [9, 10] and drug addiction [11, 12].

In addition to modulating the conditions noted above, a growing body of evidence has shown that blockade of DNA methylation can affect pain behaviors [13–15]. For example, chronic constriction injury increased the spinal global DNA methylation in rats, while intrathecal injection of the DNA methyltransferase (DNMT) inhibitor 5-azacytidine reversed this up-regulation and simultaneously attenuated the mechanical allodynia and thermal hyperalgesia [14]. Similarly, Viet et al. reported that treatment with agents promoting DNA demethylation resulted in mechanical and thermal antinociception in a mouse oral cancer model. The behavioral changes were correlated with mu-opioid receptor expression in the tumor tissue and associated neurons [15]. Consistent with these data, administration of a DNMT inhibitor reversed the hypermethylation of the mu-opioid receptor gene (*Oprm1)* and improved the analgesic effect of morphine treatment in a neuropathic pain model [16]. Furthermore, DNA methylation regulates several additional pain and analgesia-related genes in various pain models [16–18].

Clinically, chronic opioid administration is associated with increased DNA methylation at the LINE-1 global methylation site, which is correlated with pain severity [19]. Chronic stress was discovered to be associated with up-regulation of DNMT1-associated methylation of the cannabinoid receptor 1 (*Cnr1)* promoter and reduced CNR1 expression in DRG mediated chronic stress-induced increases in visceral pain [13]. Qi et al. demonstrated that demethylation of CpG sites of the cystathionine-[beta]-synthetase (*Cbs)* gene promoter region caused increased hydrogen sulfide production in DRG samples and contributes to inflammatory pain in rats [17]. Despite these discoveries, there is little information available concerning how DNA methylation might regulate postoperative pain.

The goal of our study was to identify the role of DNA methylation in modulating pain after incision. In addition to identifying the behavioral effects of a DNMT inhibitor, we sought to identify and confirm methylation-regulated targets contributing to incisional pain including the *Oprm1* gene and possibly others.

## Materials and Methods

### Animal use

All experimental protocols were reviewed and approved by Veterans Affairs Palo Alto Healthcare System Institutional Animal Care and Use Committee prior to beginning the work. All protocols conform to the guidelines for the study of pain in awake animals as established by the International Association for the Study of Pain. Male mice 8–9 weeks old of the C57BL/6J strain (weighting 20–25 gram) were obtained from Jackson Laboratory (Bar Harbor, ME). Mice were housed four per cage and maintained on a 12-h light/dark cycle and an ambient temperature of 22 ± 1°C, with food and tap water available *ad libitum*. For behavior tests and paw thickness measurement, mice were randomized into four groups (n = 6–8); for methylation measurement and genes expression, mice were randomized into two groups (n = 5–6).

### Hindpaw incision

The hindpaw incision model in mice was performed in our laboratory as described in previous studies [20, 21]. Briefly, mice were anesthetized using isoflurane 2–3% delivered through a nose cone. After sterile preparation with alcohol, a 5 mm longitudinal incision was made with a number 11 scalpel on the plantar surface of the right hindpaw. The incision was sufficiently deep to divide deep tissue including the plantaris muscle longitudinally. After controlling bleeding, a single 6–0 nylon suture was placed through the midpoint of the wound and antibiotic ointment was applied. Mice used in these experiments did not show evidence of infection in the paws at the time of behavioral or biochemical assays.

### Drug administration

5-AZA-CdR (5-Aza-2′-deoxycytidine)(Sigma-Aldrich, St. Louis, MO) was freshly dissolved in 0.9% saline (Sigma). The concentration was adjusted to 40 μg/100 μl so that a 4 mg/kg dose could be administrated intraperitoneally in a volume of 100 μl/10g body weight. Mice received either 5-AZA-CdR solution or matching vehicle 24h and 2h prior to incision and once daily for 3 days after incision. The opioid receptor antagonist naloxone hydrochloride dehydrate (Sigma-Aldrich) was freshly dissolved in 0.9% saline. Mice received either naloxone (10μg) or vehicle intraplantar on day 3 after incision in a volume of 15μl. The dosage selection of 5-AZA-CdR is based on the finding that at this dose it effectively reduced DNA methylation and produces little toxicity in mice in other experiments [22, 23].

### Nociceptive testing

All nociceptive testing was done with the experimenter blind to drug treatment.

#### Mechanical hypersensitivity

Mechanical nociceptive thresholds were assayed using von Frey filaments according to a modification of the “up-down” algorithm described by Chaplan et al [24], as described previously [20, 21]. Mice were placed on wire mesh platforms in clear cylindrical plastic enclosures of 10 cm diameter and 30 cm height. After 20 minutes of acclimation, fibers of sequentially increasing stiffness with initial bending force of 0.2 gram were applied to the plantar surface of the hindpaw adjacent to the incision, just distal to the first set of foot pads and left in place 5 sec with enough force to slightly bend the fiber. Withdrawal of the hindpaw from the fiber was scored as a response. When no response was obtained, the next stiffer fiber in the series was applied in the same manner. If a response was observed, the next less stiff fiber was applied. Testing proceeded in this manner until 4 fibers had been applied after the first one causing a withdrawal response allowing the estimation of the mechanical withdrawal threshold using a curve fitting algorithm [25].

#### Thermal sensitization

Paw withdrawal response latencies to noxious thermal stimulation were measured using the method of Hargreaves et al. [26] as we have modified for use with mice 24. In this assay, mice were placed on a temperature-controlled glass platform (29°C) in a clear plastic enclosure. After 30 min of acclimation, a beam of focused light was directed towards the same area of the hindpaw as described for the von Frey assay. A 20 s cutoff was used to prevent tissue damage. The light beam intensity was adjusted to provide an approximate 10s baseline latency in control mice. Three measurements were made per animal per test session separated by at least one minute.

### Paw edema

A laser (4381 Precicura, Limab, Goteborg, Sweden) sensor technique was used to measure the dorsal-ventral thickness of the hindpaw [27]. After induction of rapid and brief anesthesia with isoflurane, the mouse was held vertically with hindpaw resting on a table top under the laser. By applying a small metal rod to the top of ankle joint, the paw was gently held flat on the table. Using optical triangulation, a distance measuring sensor (200 mm range, 0.01 mm resolution) was used to determine the difference of the distance from the top of the hindpaw to the table top (dorsal-ventral paw thickness). Three measurements were made per paw per animal.

### RNA isolation and real-time quantitative polymerase chain reaction (PCR) amplification

Mice were first euthanized by carbon dioxide asphyxiation and an ovular full-thickness patch of skin providing 1.5- to 2-mm margins surrounding the hindpaw incisions was collected. Spinal cord tissue was harvested by extrusion. Lumbar spinal cord segments were dissected on a chilled surface. Dorsal root ganglia (DRG) (L3-S1) were dissected using low power binocular magnification as described previously [28]. Dissected tissue was then quick-frozen in liquid nitrogen and stored at -80°C until required for analysis. For real-time quantitative PCR, total RNA was isolated from skin and spinal cord using the RNeasy Mini Kit (Qiagen, Valencia, CA) according to the manufacturer's instructions. The purity and concentration were determined spectrophotometrically. The total RNA samples were reverse transcribed into complementary DNA using a First Strand complementary DNA Synthesis Kit (Invitrogen, Carlsbad, CA). Real-time PCR was performed in an ABI prism 7900HT system (Applied Biosystems, Foster City, CA). All PCR experiments were performed using the SYBR Green I master kit (Applied Biosystems). The primer sequences for 18S message RNA (mRNA) are aagacgatcagataccgtcgtag (forward) and tccgtcaattcctttaagtttca (reverse). All of the other primer sets were purchased from SABiosciences (Valencia, CA). The amplification parameters were described previously [21]. Melting curves were performed to document single product formation and agarose electrophoresis confirmed product size. All the primers were purchased from SABiosciences (SABiosciences, Valencia, CA). As negative controls, RNA samples that were not reverse transcribed were run. Data were normalized to 18S mRNA expression.

### DNA isolation and global DNA methylation

Genomic DNA was isolated with the GenElute^™^ Mammalian Genomic DNA Miniprep Kit (Sigma-Aldrich) according to manufacter's instructions. DNA was treated with RNAase to remove RNA contaminants. Isolated DNA was quantified using an ND-1000 NanoDrop spectrophotometer (Thermo Scientific, Wilmington, DE). Assessment of global DNA methylation status was accomplished by using the MethylFlash Methylated DNA Quantification Kit (Epigentek, Farmingdale, NY) according to manufacter's instructions. The methylated fraction of DNA was identified using 5-methylcytosine monoclonal antibodies and quantified by an enzyme-linked immunosorbent assay–like reaction. The levels of methylated DNA were measured at 450nm with a VERSmax Microplate Reader (Molecular Devices, Sunnyvale, CA). The percentage of 5-mC was calculated using the formula provided in the kit procedure and were normalized to percentage of control.

### Statistical analysis

All data are expressed as mean ± standard error of the mean (SEM). The time course changes of gene expression and global methylation within each group was analyzed by one-way ANOVA with post-hoc Bonferroni's testing for multiple comparisons. Comparisons between two groups for behavior test, paw thickness and gene expression were analyzed by unpaired t-test at each timepoint. *P* values less than 0.05 were considered significant (Prism 5; GraphPad Software, La Jolla, CA).

## Results

### Effects of DNMT inhibition on incision-induced nociceptive sensitization and edema

**Fig 1:**
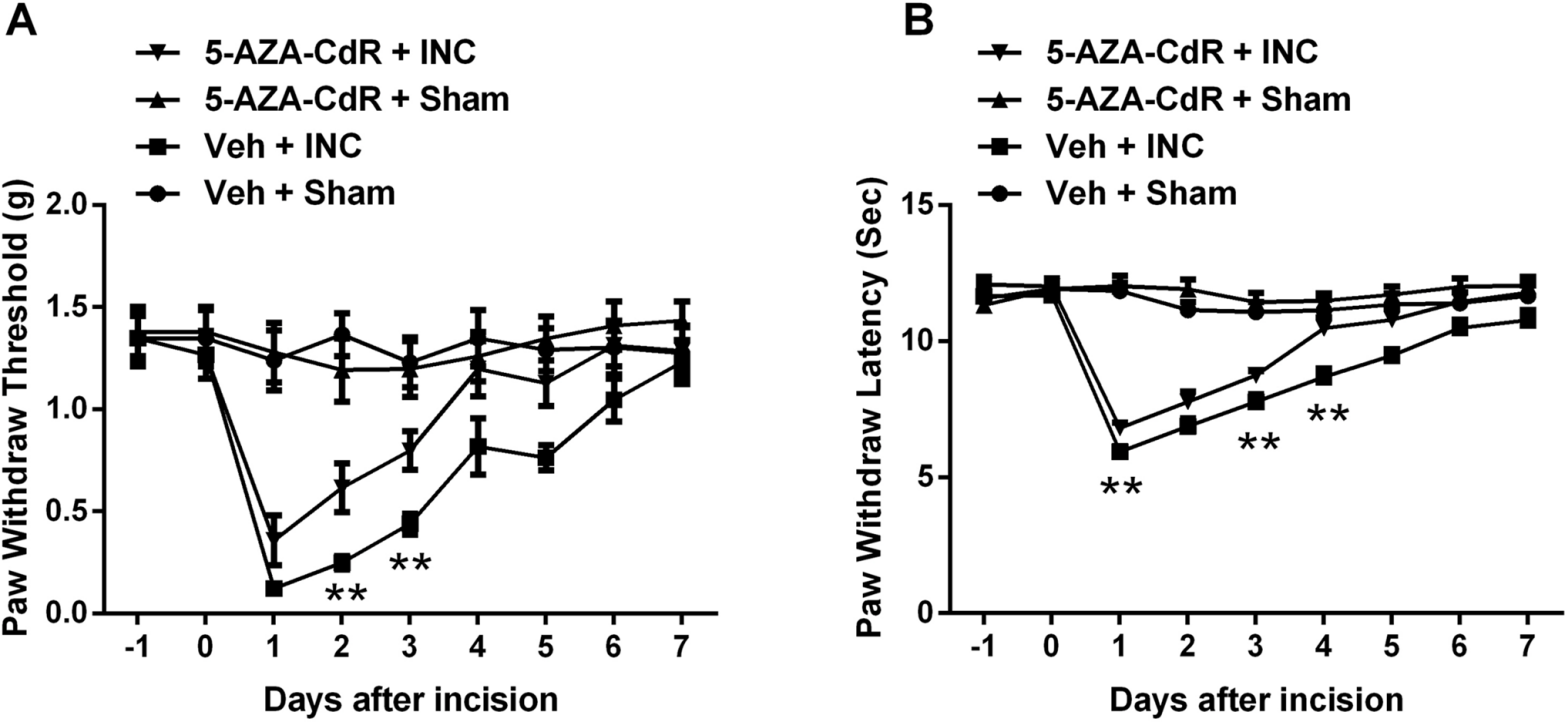
Assessment of DNMT inhibitor 5-Aza-2′-deoxycytidine on incision-induced mechanical and thermal sensitization. Blocking DNMT attenuated incision-induced mechanical hypersensitivity (A) and thermal sensitization (B). Animals received intraperitoneal injection of 5-Aza-2′-deoxycytidine (4 mg/kg) or vehicle (saline) 24h and 2h prior to incision and once daily for 3 days after incision. Values are displayed as the mean ± SEM. N = 8. **p<0.01, 5-AZA-CdR + INC group vs. Veh + INC group. Veh = vehicle; INC = incision; 5-AZA-CdR = 5-Aza-2′-deoxycytidine.

To determine whether DNA methylation alters incision-induced sensitization, the DNMT inhibitor 5-AZA-CdR was administrated daily to incised mice. Fig 1A and 1B present data demonstrating that systemic administration of 5-AZA-CdR significantly attenuated incision-induced mechanical hypersensitivity and thermal sensitivity. 5-AZA-CdR had no effect on the nociceptive thresholds of control animals (Fig 1).

**Fig 2:**
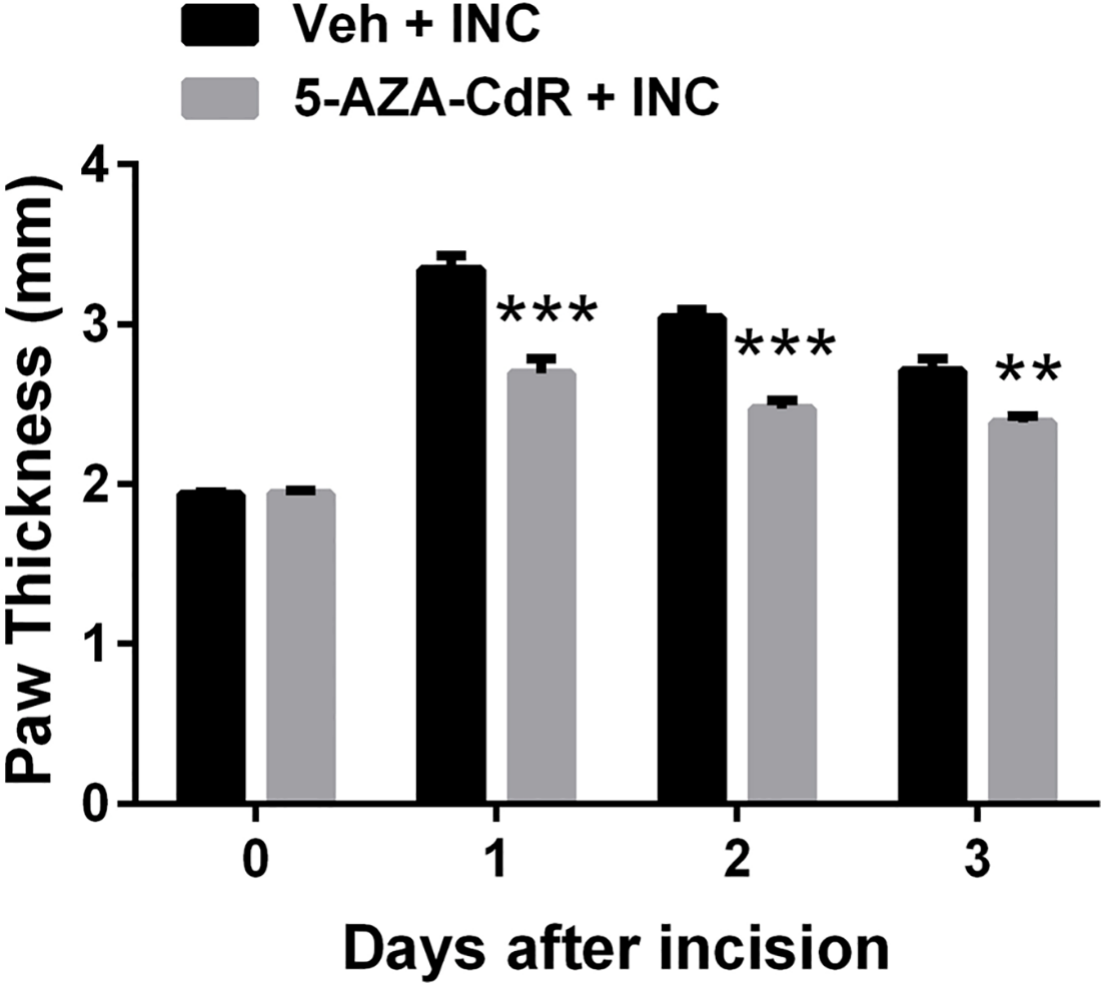
Effect of DNMT inhibitor 5-Aza-2′-deoxycytidine on paw edema induced by incision. 5-Aza-2′-deoxycytidine significantly reduced incision-induced hindpaw edema. Values are displayed as the mean ± SEM. N = 8. ** p<0.01, *** p<0.001, vs. vehicle treated group. Veh = vehicle; INC = incision; 5-AZA-CdR = 5-Aza-2′-deoxycytidine.

To determine the ability of 5-AZA-CdR treatment to reduce indices of the inflammatory response in incised animals, we measured changes in paw thickness at time points up to 3 days post-incision. 5-AZA-CdR significant reduced incision-induced edema at the 1–3 day time points (Fig 2).

### Alteration of DNMT expression and global DNA methylation after incision

**Fig 3:**
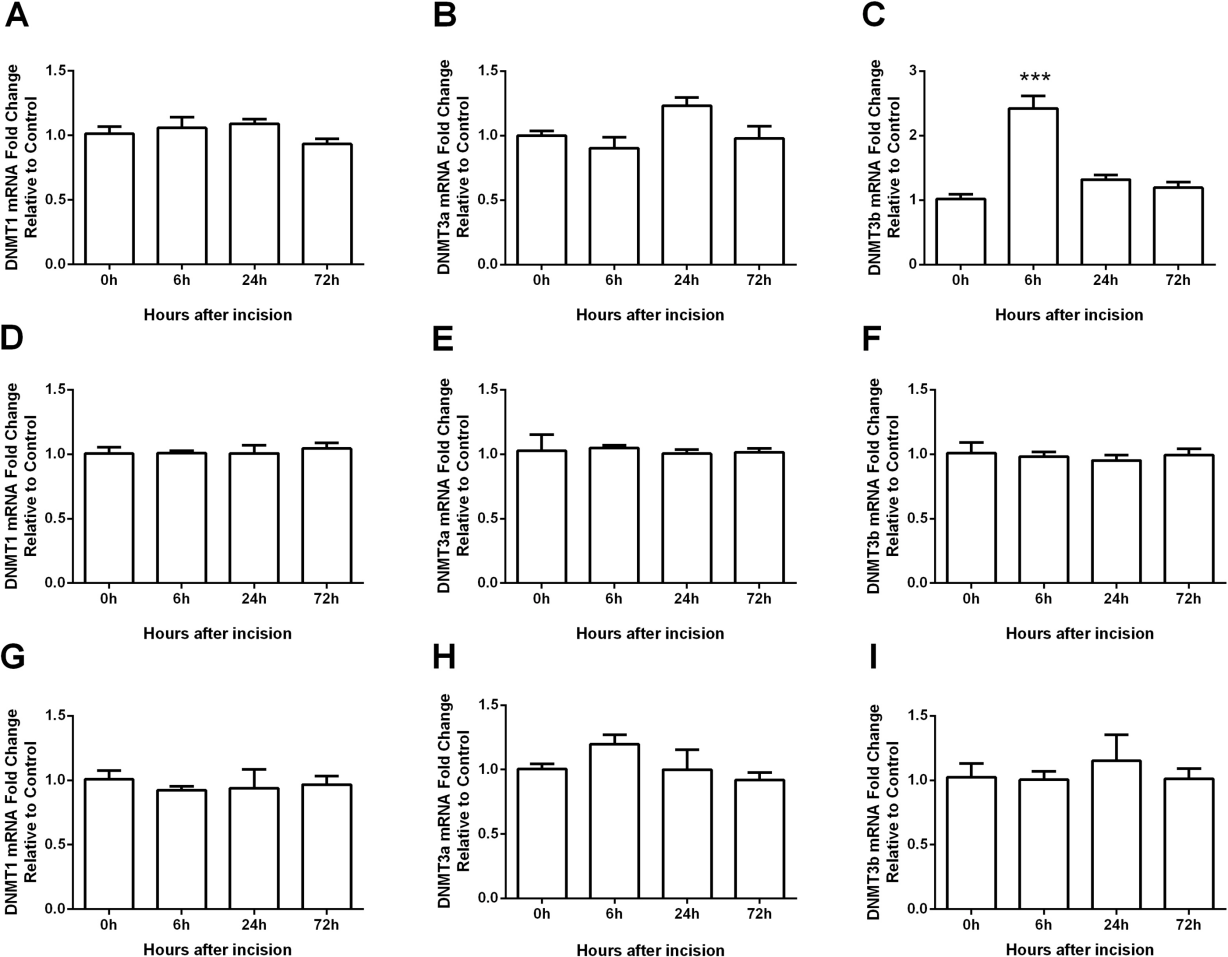
Expression of DNMTs in skin (A-C), spinal cord (D-F) and DRG (G-I) tissues after incision. The mRNA level of DNMT3b (C) was significantly increased at 6h in skin after incision, while DNMT1 (A) and DNMT3a (B) did not change in skin. The mRNA levels of DNMT1 (D and G), DNMT3a (E and H), or DNMT3b (F and I) were not altered in spinal cord or DRG after incision, respectively. Values are displayed as the mean ± SEM, n = 6. ***p<0.001 vs. day 0 (before incision).

Because a DNMT inhibition significantly attenuated incision-induced nociceptive sensitization, we hypothesized that pro-nociceptive changes may be associated with enhanced DNA methylation. To test this idea we first examined the expression of DNA methyltransferases (DNMTs) in incised skin and spinal cord tissue. The mammalian genome encodes three active DNMTs: DNMT1, DNMT3a and DNMT3b. DNMT1 is described as the maintenance methyltransferase, while DNMT3a and DNMT3b are methyltransferases often expressed *de novo* [29, 30]. We found that the mRNA level of DNMT3b was significantly increased (6h after incision in skin tissue), and the mRNA levels of DNMT1 and DNMT3a were not changed in skin tissue (Fig 3A–3C). In addition, none of the DNMTs’ expression was altered in the spinal cord (Fig 3D–3F) or DRG (Fig 3G–3I).

**Fig 4:**
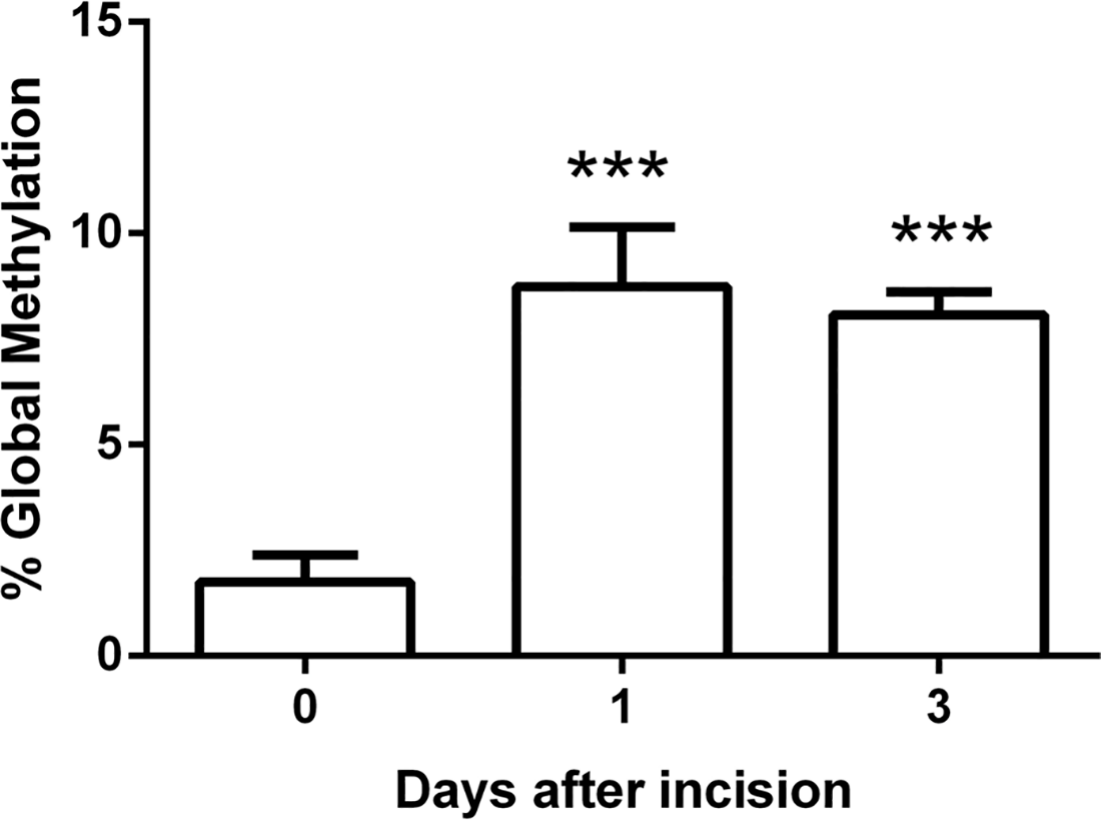
The changes of global DNA methylation after incision. The global DNA methylation was increased in skin at day 1 and day 3 after incision. Values are displayed as the mean ± SEM, n = 5. ***p<0.001 vs. day 0 (before incision).

Since DNMT3b mRNA level was upregulated in skin after incision, we examined global DNA methylation in skin and found that the global DNA methylation was increased in skin tissue at day 1 and day 3 after incision (Fig 4). Additionally, we also examined the global methylation in spinal cord and no changes were found in spinal cord tissue across this time course (data not shown).

### Effects of 5-AZA-CdR on anti-inflammatory cytokine gene expression

**Fig 5:**
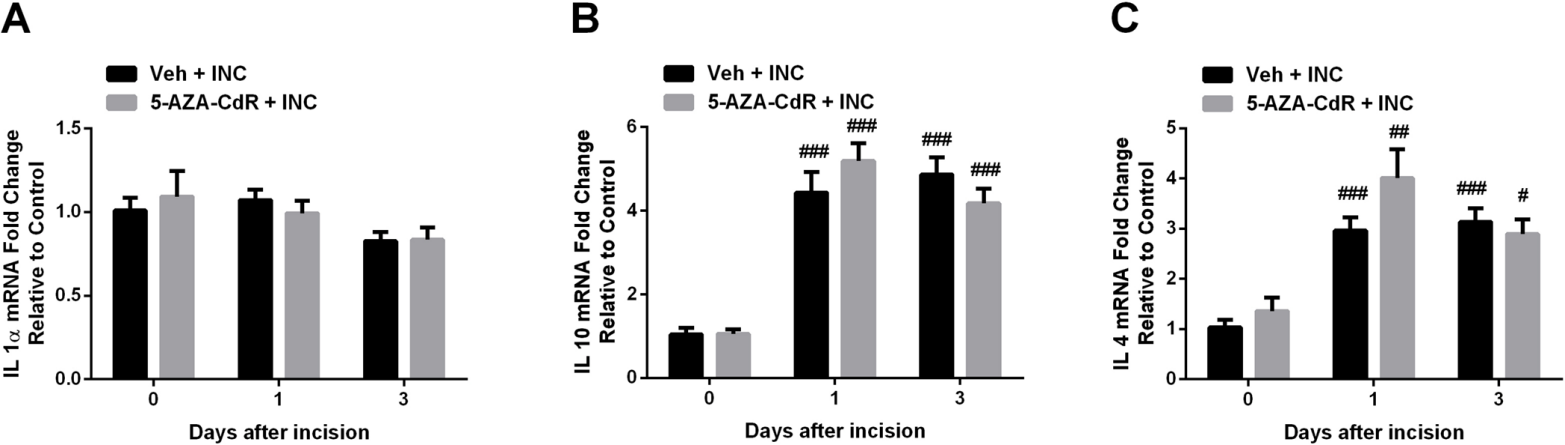
Effect of DNMT inhibitor 5-Aza-2′-deoxycytidine on Il-1α (A), Il-4 (B) and Il-10 (C) mRNA levels in skin tissue after incision. Incision induced the up-regulation of Il-4 and Il-10 expression, however, DNMT inhibitor failed to alter Il-1α (A), Il-4 (B) and Il-10 (C) expression compared with the vehicle treated group. Values are displayed as the mean ± SEM. N = 5. # p<0.05, ## p<0.01, ### p<0.001 vs. day 0 (before incision). Veh = vehicle; INC = incision; 5-AZA-CdR = 5-Aza-2′-deoxycytidine.

DNMT inhibition leads to the demethylation of gene promoters and subsequent gene activation. Here, we examined the expression of *Il-1α*, *Il-4* and *Il-10* mRNA levels after incision under 5-AZA-CdR treatment, because these anti-inflammatory cytokines have been demonstrated to have antinociceptive activities in various pain models [31–33], and are regulated via DNA methylation [34–36]. Both *Il-10* and *Il-4* genes were upregulated after incision. However, expression of none of these genes was altered by 5-AZA-CdR treatment (Fig 5).

### Effects of 5-AZA-CdR on mu-opioid receptor and endogenous opioid gene expression

**Fig 6:**
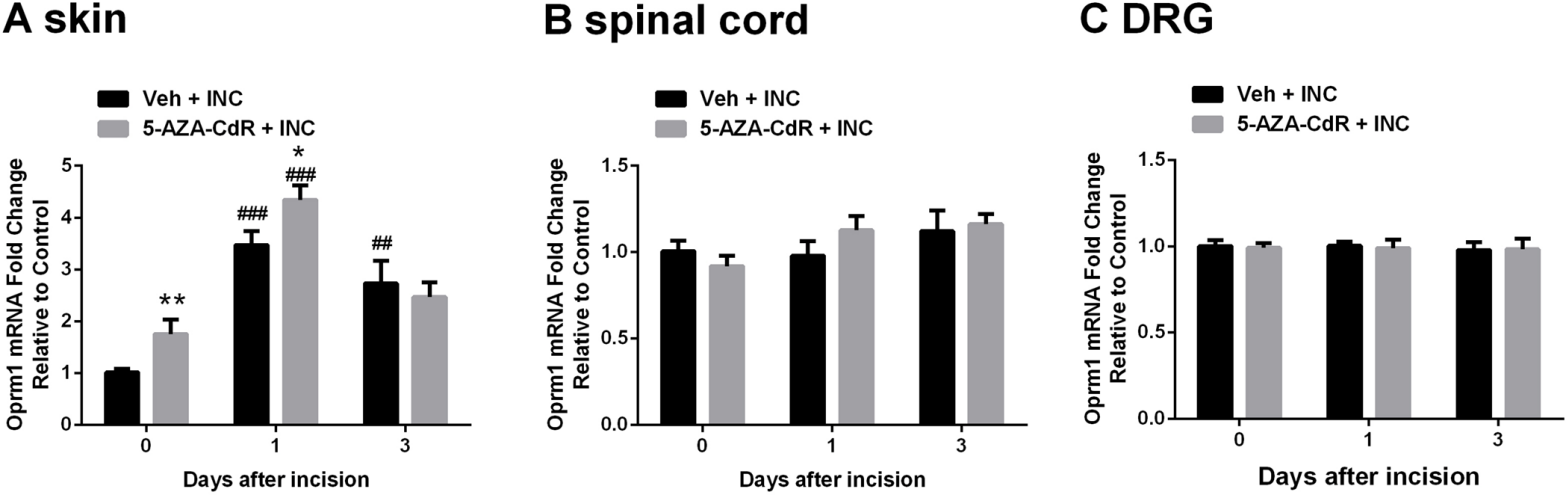
Changes in mu-opioid receptor mRNA (*Oprm1)* expression after incision. (A) The mRNA level of *Oprm1* significantly increased after incision in skin, and further increased in mice treated with 5-Aza-2′-deoxycytidine before and at day 1 after incision. The mRNA level of *Oprm1* did not alter in spinal cord (B) or DRG (C) after incision. Values are displayed as the mean ± SEM. N = 6. **p<0.01, 5-AZA-CdR + INC group vs. Veh + INC group. ## p<0.01, ### p<0.001 vs. day 0 (before incision). Veh = vehicle; INC = incision; 5-AZA-CdR = 5-Aza-2′-deoxycytidine.

Activation of peripheral or central mu-opioid receptors can produce analgesic effects in various animal pain models and humans [16, 37, 38]. Furthermore, mu-opioid receptor expression was previously demonstrated to be epigenetically regulated via DNA methylation [16, 19, 39]. Therefore, we examined the peripheral and central *Oprm1* expression after incision and/or DNMT inhibitor treatment. The mRNA level of *Oprm1* was increased in skin after incision and further increased in mice treated with 5-AZA-CdR (Fig 6A). Additionally, 5-AZA-CdR itself caused a transient upregulation of *Oprm1* mRNA expression in skin at day 0 (Fig 6A), without significant changes at day 1 and day 3 (data not shown), which indicated that 5-AZA-CdR regulates Oprm1 gene expression in the control and incision settings. Neither incision nor 5-AZA-CdR altered the mRNA levels of *Oprm1* in spinal cord (Fig 6B) or DRG (Fig 6C).

**Fig 7:**
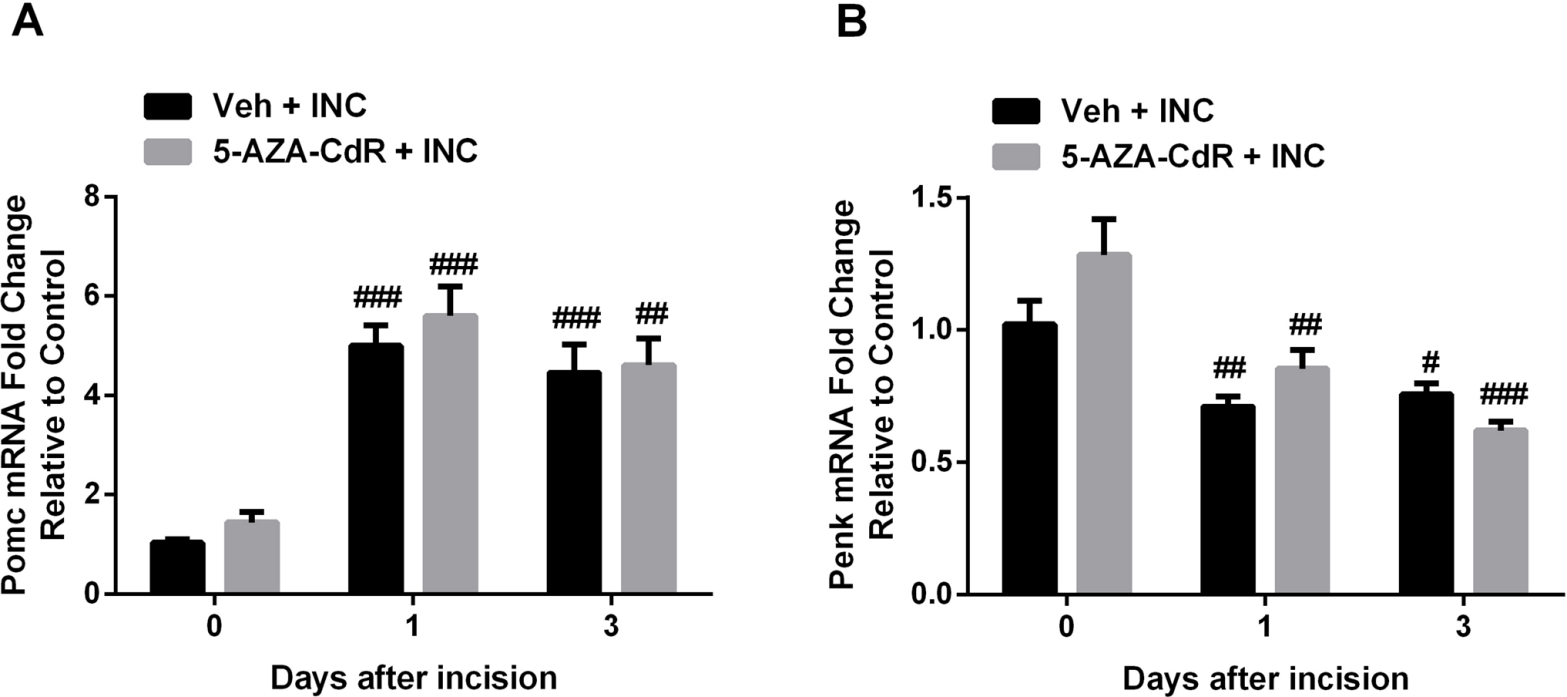
Changes in endogenous opioid peptides encoding genes, proopiomelanocortin (POMC) and proenkephalin (PENK) mRNA levels in skin after incision. (A) The mRNA level of POMC significantly increased after incision in skin. (B) The mRNA level of PENK significantly decreased after incision in skin. Values are displayed as the mean ± SEM. N = 6. # p<0.05, ## p<0.01, ### p<0.001 vs. day 0 (before incision). Veh = vehicle; INC = incision; 5-AZA-CdR = 5-Aza-2′-deoxycytidine.

Next, since only peripheral *Oprm1* expression was changed after incision and DNMT inhibitor treatment, we examined the peripheral expression of endogenous opioid peptide encoding genes, proopiomelanocortin (*Pomc*) and proenkephalin (*Penk*). As shown in Fig 7, incision induced the upregulation of *Pomc* mRNA expression in skin and the downregulation of *Penk* mRNA levels, however, 5-AZA-CdR failed to alter *Pomc* and *Penk* expression compared with vehicle treated group.

We examined the expression of other methylation-regulated pain-related genes in skin and spinal cord tissues, including microRNA203 [20, 40], encoding the endothelin B receptor (EDNRB) [41, 42], galanin [43, 44] and dipeptidyl peptidase 4 [45, 46]. However, none of them showed epigenetic regulation under DNMT inhibitor treatment (data not shown).

### Effects of intraplantar injection of opioid antagonist, naloxone on 5-AZA-CdR attenuated incisional pain

**Fig 8:**
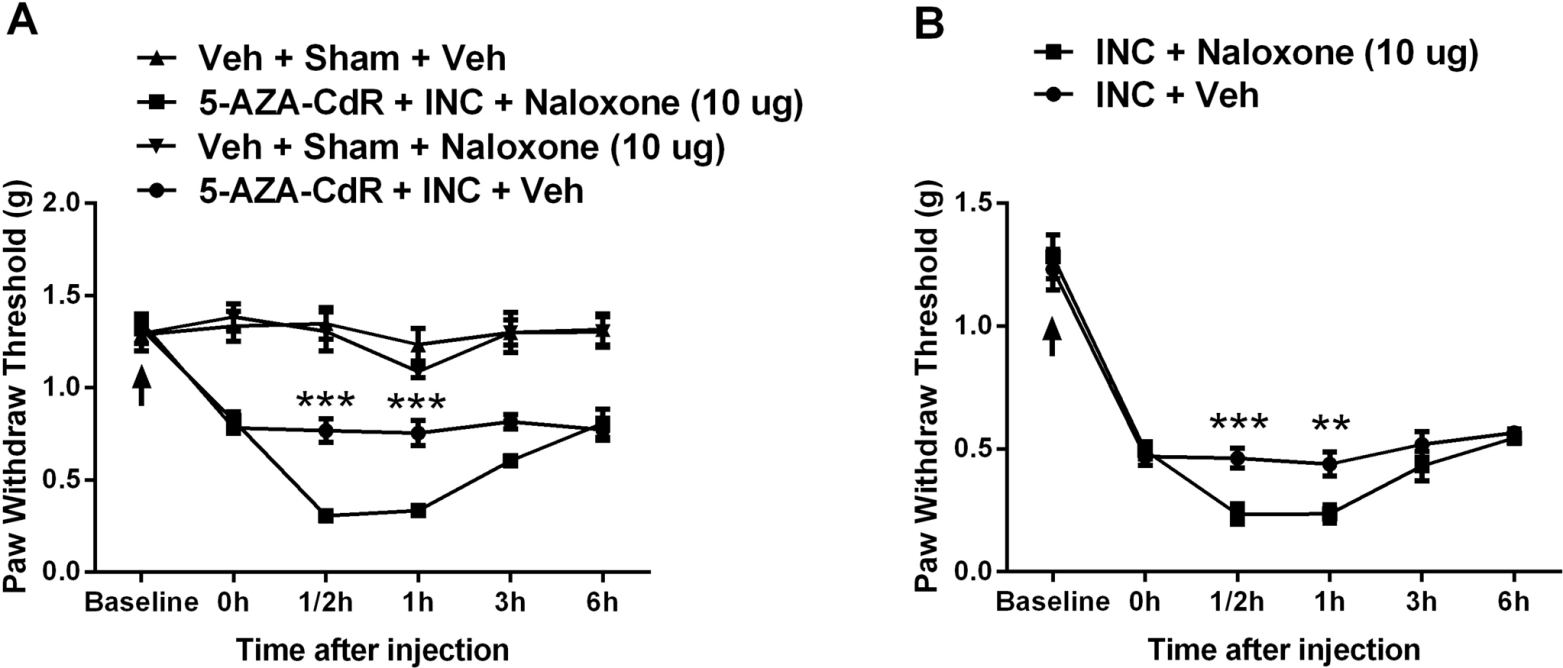
Assessment of peripheral mu-opioid receptor signaling on nociceptive sensitization after incision. Intraplantar injection of opioid receptor antagonist naloxone at day 3 after incision significantly reversed 5-Aza-2′-deoxycytidine attenuated, incision-induced mechanical hypersensitivity (A), and significantly exacerbated incisional mechanical hypersensitivity as well (B). Arrow indicates the time point for incision operation. Values are displayed as the mean ± SEM, n = 6, ** p<0.01, *** p<0.001, 5-AZA-CdR + INC + Naloxone group vs 5-AZA-CdR + INC + Veh group. Veh = vehicle; INC = incision; 5-AZA-CdR = 5-Aza-2′-deoxycytidine.

Since *Pomc* and *Oprm1* were up-regulated in skin tissue after incision and (for *Oprm1*) further increased in mice treated with 5-AZA-CdR, we determined whether the peripheral mu-opioid receptor signaling pathway was involved in nociceptive sensitization after incision. Peri-incisional administration of the opioid receptor antagonist naloxone (10 μg intraplantar) at day 3 after incision significantly exacerbated mechanical hypersensitivity consistent with an active process of local opioid analgesia (Fig 8A). Furthermore, local injection of naloxone (10 μg) significantly reduced threshold to the same level observed in the inhibitor treated mice (Fig 8B). This is consistent with opioid tone being involved in mediating the effects of methylation after incision. Naloxone did not have any effect on the nociceptive thresholds of non-incised control animals (Fig 8A).

## Discussion

Over the past decade, a significant amount of work has been done to elucidate the mechanisms involved in nociceptive sensitization after surgical incision. Recently, attention has to a degree shifted toward understanding how epigenetic mechanisms regulate postoperative pain [21, 47, 48]. DNA methylation is a key epigenetic mechanism controlling transcription factor binding and gene expression. However, the role of DNA methylation with regard to postoperative pain has not yet been explored. In this study, our principal observations were: (1) Blockade of DNMT attenuated incision-induced nociceptive sensitization as well as incision-induced edema. (2) Global DNA methylation and DNMT3b expression were increased in skin after incision, but none of DNMT1, DNMT3a or DNMT3b was altered in spinal cord or DRG after incision. (3) The expression of *Pomc* encoding β-endorphin and *Oprm1* encoding mu-opioid receptor was upregulated peripherally after incision; moreover, *Oprm1* was further increased under DNMT inhibitor treatment. (4) Peripheral injection of opioid receptor antagonist, naloxone significantly exacerbated incision-induced mechanical hypersensitivity. Together these observations suggest that DNA methylation is functionally relevant to incisional nociceptive sensitization, and that peripheral mu-opioid receptor signaling might be one DNA methylation regulated pathway controlling sensitization after incision.

Available reports from the literature are largely similar in their findings that DNMT inhibition provides antinociceptive effects [13–15]. 5-Azacytidine and its more selective deoxy analog, 5-aza-2-deoxycytidine (5-AZA-CdR), are well-known DNA methylation inhibitors, and both have been approved by the US food and Drug Administration for the treatment of myelodysplastic syndrome [49]. Both drugs inhibit the DNMTs’ activity by substituting for cytosine residues and forming covalent bonds with the DNMTs, resulting in demethylation [49]. A previous report showed that the systemic administration of 5-AZA-CdR produced significant antinociception to mechanical stimuli in the mice oral cancer model [15]. Spinal cord methylation was suggested to support nociception; intrathecal injection of 5-azacytidine attenuated mechanical and thermal sensitivity in chronic constriction injury-induced neuropathic pain model [14]. Additionally, knockdown of DNMT1expression prevented chronic stress induced increases in visceral pain [13]. Our data are functionally consistent with all of these observations in that 5-AZA-CdR reduced incision-induced mechanical and thermal sensitivity. However, since 5-AZA-CdR treatment started before incision and continued after, it is unclear at what specific time point DNMT inhibition might be most effective. Additionally, our data also showed that 5-AZA-CdR reduced incision-induced edema. However, since the levels of several pain-related anti-inflammatory cytokines were unchanged by DNMT inhibition, the mechanisms for the effects on edema are unclear.

DNMTs transfer a methyl group from S-adenosyl-methionine to the fifth carbon of cytosine residues in the genomic CpG dinucleotides. DNMT1, DNMT3a, DNMT3b are the principle isoforms that establish and maintain the patterns of DNA methylation in mammals [50]. Generally, DNMT1 is described as the maintenance methyltransferase, copying methylation marks throughout cell division [29]. DNMT3a and DNMT3b are de novo methyltransferases, methylating specific DNA marks in response to environmental factors [30]. It has been observed that persistent pain alters the expression of DNMTs. For example, CFA-induced inflammatory pain caused the global upregulation of DNMTs in spinal cord dorsal horn, while spared nerve injury lead to the downregulation of DNMT1 in spinal cord [51]. More recently, Pollema-Mays et al. using the spared nerve injury model found that the DNMT1 and DNMT3a were moderately upregulated in DRG, and DNMT3b showed a robust increase in DRG [52]. In our present study, we only found DNMT3b to be upregulated after incision, while none of DNMTs was changed in spinal cord. Baubec et al. observed that DNMT3b has a prominent genome-wide role in controlling transcription by selectively binding to the bodies of transcribed genes leading to their preferential methylation [53]. Interesting, among the DNMTs, the DNMT3B has the most alternatively spliced isoforms with about 40 different DNMT3B isoforms identified so far which influence the functional properties of this enzyme [54, 55]. It is not clear which of these variants is operative in our model.

Global methylation is a measure of the overall state of the DNA methylation and has long-range consequences on genome function [56, 57]. Consistent with the up-regulation of DNMT expression in peri-incisional tissue, we also observed the increased global DNA methylation. There were no such changes measured in spinal cord. Therefore, the changes in global DNA methylation and DNMT expression appear to be tissue- and disease-specific in response to various types of injuries. Similarly, it has been reported that chronic peripheral nerve injury caused decreases in global methylation in prefrontal cortex and amygdala, but not in thalamus and visual cortex [58]. Furthermore, overall tissue methylation levels do not always correlate with methylation changes at the local level. For example, Meller et al reported global methylation was reduced following preconditioning ischemia in primary neuronal culture; however, selective chromosomes showed enhanced hypermethylation [59]. In addition, opioid receptor and endogenous gene expression are regulated by the other epigenetic mechanisms, such as histone acetylation and microRNA regulation [60, 61]. Our observations were that Oprm1 expression actually increased after incision despite increased global skin methylation levels, but blockade of DNMT further increased Oprm1 expression. This suggests methylation limits or counterbalances other incision-induced processes.

Accumulating evidence shows that the expression of mu-opioid receptor and its relevant ligands are altered in peripheral and central nervous system tissue in various pain models and contribute to regulating nociceptive sensitization [37, 38, 62, 63]. Furthermore, this receptor is epigenetically regulated via DNA methylation [16, 19, 64]. In general, hypermethylation of gene promoters is associated with transcriptional repression by preventing the binding of transcription factors for their binding sites, whereas, hypomethylation results in gene activation [50]. For example, Zhou et al. observed that peripheral nerve injury caused a decreased mu-opioid receptor expression with an increased methylation status of *Oprm1* gene promoter in DRG tissue. Treatment with the DNMT inhibitor 5-AZA-CdR reversed the decreased *Oprm1* expression via demethylation of its promoter and improved the analgesic effects of morphine administration [16]. In the current study, we showed that both *Oprm1* and its ligand *Pomc* were significantly increased in skin after incision, and *Oprm1* gene expression was further increased when DNMT was blocked. Pharmacological experiments showed that intraplantar injection of an opioid receptor antagonist exaggerated 5-AZA-CdR-attenuated mechanical allodynia after incision, suggesting that the up-regulation of peripheral mu-opioid receptor leads to the decreased nociceptive sensitivity. Although mu-opioid receptor is generally considered to exert its anti-nociceptive actions within the CNS [65], peripheral mu-opioid receptor has become increasingly recognized as important for limiting sensitization in the settings of tissue injury and inflammation [66]. For example, it has been reported that *Oprm1* is expressed in nerve terminals and keratinocytes in skin as well as on immune cells [67–69]. Furthermore, our previous studies demonstrated that endogenous opioid peptides (β-endorphin and others) were produced by neutrophils after incision [70]. Therefore, the upregulation of *Oprm1* expression under 5-AZA-CdR treatment after incision may sensitize local or infiltrating cells to the effects of endogenous (or perhaps exogenous) opioid ligands. The reduction in edema similarly suggested local opioid receptor mediated anti-inflammatory effects. Additional studies have demonstrated that expression of pain-related genes microRNA203 [20, 40], encoding the endothelin B receptor (EDNRB) [41, 42], galanin [43, 44] and dipeptidyl peptidase 4 [45, 46] are also under the control of methylation-related epigenetic mechanisms. However, DNMT inhibition failed to alter their expression in setting of our incisional pain model.

In Summary, the present study is the first to show that incision induces changes in global DNA methylation and DNMT expression in skin, and suggests that regulation of DNA methylation can control nociceptive sensitization after incision. DNMT inhibition reduced incisional mechanical hypersensitivity which appears to be due at least in part to the up-regulation of *Oprm1* expression. It is widely believed that large numbers of genes regulate nociception; DNA methylation can broadly control gene expression in setting of tissue injury potentially providing an overarching mechanism controlling some portion of this response [71–73]. Therefore, our theory that a DNMT inhibitor attenuated incisional sensitization via increasing *Oprm1* expression likely explains only a portion of the inhibitor’s effects. Future studies might include the investigation of DNA methylation status of *Oprm1* gene under DNMT regulation, as well as other groups of genes, e.g. by using array or sequencing technologies, and more broadly addressing the roles of DNMTs by using selective DNMT inhibitors or conditional knockdown strategies in the incisional pain model.
